# Fcγ Receptor Heterogeneity in Leukocyte Functional Responses

**DOI:** 10.3389/fimmu.2017.00280

**Published:** 2017-03-20

**Authors:** Carlos Rosales

**Affiliations:** ^1^Departamento de Inmunología, Instituto de Investigaciones Biomédicas, Universidad Nacional Autónoma de México, Ciudad de México, Mexico

**Keywords:** immunoglobulin, antibody, phagocytosis, neutrophil, ERK, NF-κB, integrin

## Abstract

Antibodies participate in defense of the organism from all types of pathogens, including viruses, bacteria, fungi, and protozoa. IgG antibodies recognize their associated antigen *via* their two Fab portions and are in turn recognized though their Fc portion by specific Fcγ receptors (FcγRs) on the membrane of immune cells. Multiple types and polymorphic variants of FcγR exist. These receptors are expressed in many cells types and are also redundant in inducing cell responses. Crosslinking of FcγR on the surface of leukocytes activates several effector functions aimed toward the destruction of pathogens and the induction of an inflammatory response. In the past few years, new evidence on how the particular IgG subclass and the glycosylation pattern of the antibody modulate the IgG–FcγR interaction has been presented. Despite these advances, our knowledge of what particular effector function is activated in a certain cell and in response to a specific type of FcγR remains very limited today. On one hand, each immune cell could be programmed to perform a particular cell function after FcγR crosslinking. On the other, each FcγR could activate a particular signaling pathway leading to a unique cell response. In this review, I describe the main types of FcγRs and our current view of how particular FcγRs activate various signaling pathways to promote unique leukocyte functions.

## Introduction

The first antibodies produced by the adaptive immune response belong to the immunoglobulin M (IgM) class. These antibodies present low affinity for pathogen antigens. However, as the adaptive immune response progresses, antibodies produced are mainly of the IgG class. These antibodies present higher affinity and greater specificity for their particular antigen. Thus, IgG antibodies are involved in protection from all types of pathogens, including viruses, bacteria, fungi, and protozoa (1).

Although, IgG molecules are key for controlling infections, these antibodies usually do not directly damage the microorganisms they recognize. Nowadays, it is well known that leukocytes of the innate immune system are responsible for the protective effects of these antibodies. Some antibodies can directly neutralize toxins or viruses, and activate complement. By binding to a toxin, antibodies prevent the toxin from reaching its receptor on a cell and thus protect the cell. Similarly, by binding to a virus, antibodies inhibit uncoating of the virus and prevent a productive viral infection (2). Antibodies can also activate complement, which is then deposited on pathogens to promote phagocytosis *via* complement receptors (3, 4), or to induce bacterial lysis *via* the formation of the membrane attack complex (5).

IgG antibodies recognize their associated antigen *via* their two Fab (fragment antigen-binding) portions and are in turn recognized though their Fc (fragment crystallizable) portion by specific Fcγ receptors (FcγRs) on the membrane of immune cells (6, 7). Crosslinking of FcγR on the surface of cells activates several effector functions. These effector functions are aimed toward the destruction of pathogens and the induction of an inflammatory response that is beneficial during infections (8). Depending on the cell type, and also on the Fcγ receptor type, these effector functions include phagocytosis, activation of the oxidative burst, cell degranulation, antibody-dependent cell-mediated cytotoxicity (ADCC), and activation of genes for production of cytokines and chemokines (8, 9).

Because FcγR-mediated cell effector functions vary considerably among different leukocytes and types of IgG, it is then of great interest to understand how a certain FcγR is activated to induce a particular cellular function. This knowledge would help us in the future to augment an effective anti-microbial response for example during infections, or to inhibit an exacerbated inflammatory or autoimmune response (10, 11). In addition, it will help us to develop new therapeutic antibodies capable of interacting with certain Fc receptors to induce particular effector cell functions (12). The first level of control is clearly the binding of IgG molecules to FcγRs. In the past few years, the binding of IgG molecules to FcγRs has been examined more carefully, and new evidences on the manner some factors modulate the IgG–FcγR interaction have been described. These factors include the particular IgG subclass (13, 14) and the glycosylation pattern of the antibody (15–17).

Despite these advances on how IgG molecules and FcγRs interact, our knowledge of what particular effector function is activated in a certain cell and in response to a specific type of FcγR remains very limited today. The traditional view has been that each immune cell could be programmed to perform a particular cell function after FcγR crosslinking. Another more recent view is that each FcγR activates a particular signaling pathway leading to a unique cell response. In this review, I describe the main types of FcγRs, and the recent evidence that supports the idea that a specific FcγR induces a unique cell response.

## Fcγ Receptors

**Figure 1:**
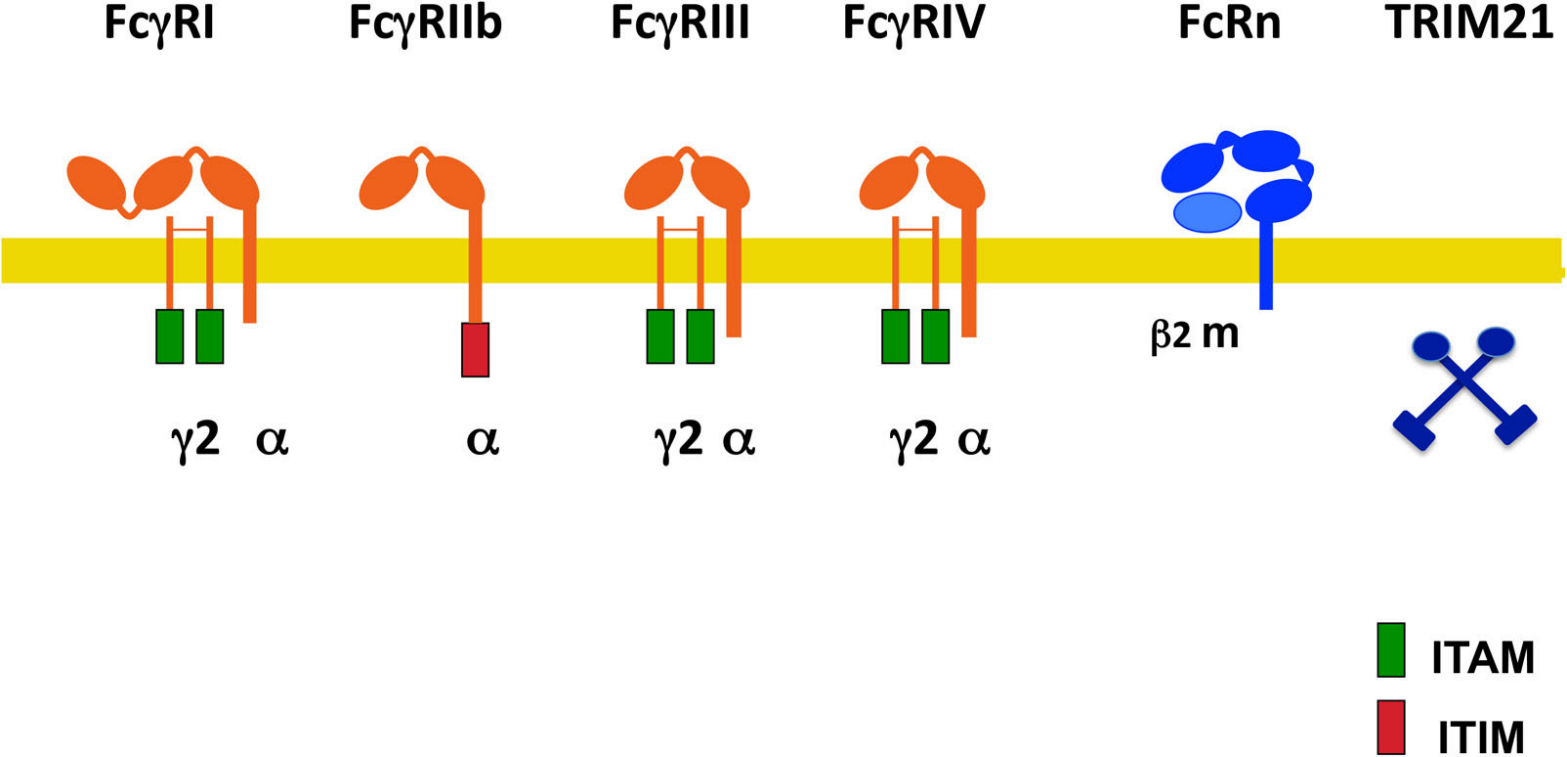
**Mouse Fcγ receptors (FcγRs)**. Schematic illustration of mouse receptors for IgG. FcγRs are shown relative to the cell membrane (yellow line) and together with their respective signaling subunits. γ2, FcR gamma subunit dimer; β2m, beta-2 microglobulin; ITAM, immunoreceptor tyrosine-based activation motif (green rectangle); ITIM, immunoreceptor tyrosine-based inhibition motif (red rectangle); FcRn, neonatal Fc receptor. TRIM21 is a cytosolic receptor.

Fcγ receptors are a family of glycoproteins expressed on the membrane of immune cells, and capable of binding the Fc portion of IgG antibody molecules (9, 14). These receptors can bind to the various IgG subclasses with different affinities (8), and when crosslinked by multivalent antigen-antibody complexes, can induce different cellular responses. In mice, there are three exclusive IgG receptors (mFcγRI, mFcRn, and mTRIM21), and three receptors that can bind both IgG and IgE (mFcγRIIb, mFcγRIII, and mFcγRIV) (18) (Figure 1). The latter dual-specific receptors prefer binding to IgG (affinity is around 2 log higher) that they are usually described as IgG receptors (18). However, interacting with IgE can also induce biological responses (19). All these receptors bind IgG on the membrane of the cells expressing them, except the neonatal FcR (mFcRn) (20, 21) and the cytosolic tripartite motif-containing protein 21 (TRIM21) (22, 23) that bind antibody molecules once internalized. In addition, polymorphisms for mouse Fc receptors have been described. Ly17.1 and Ly17.2 are alleles for mFcγRIIb, and V, T, H are alleles for mFcγRIII (Figure 1). These receptors can also be divided into activating (mFcγRI, mFcγRIII, and FcγRIV) and one inhibitory (mFcγRIIb) receptors (14, 24).

**Figure 2:**
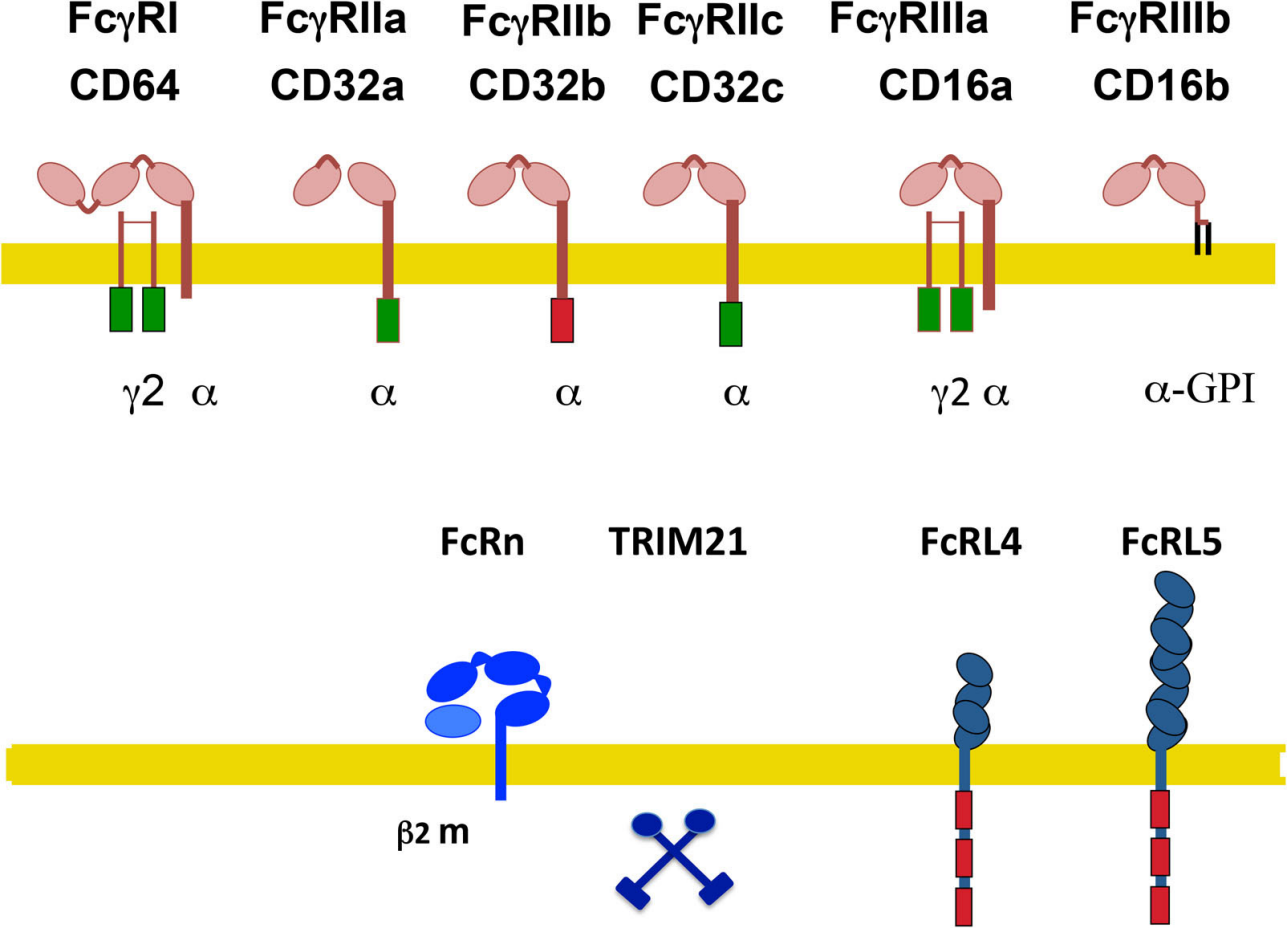
**Human Fcγ receptors (FcγRs)**. Schematic illustration of human receptors for IgG. FcγRs are shown relative to the cell membrane (yellow line) and together with their respective signaling subunits. Upper panel shows the classical FcγR (those containing typical Ig-domains). Lower panel shows the non-classical FcγR. γ2, FcR gamma subunit dimer; β2m, beta-2 microglobulin; ITAM, immunoreceptor tyrosine-based activation motif (green rectangle); ITIM, immunoreceptor tyrosine-based inhibition motif (red rectangle); FcRn, neonatal Fc receptor. TRIM21 is a cytosolic receptor. The FcR-like receptors FcRL4 and FcRL5 are inhibitory receptors that are expressed exclusively on B cells.

In humans, also several activating receptors (FcγRI/CD64, FcγRIIa/CD32a, FcγRIIc/CD32c, and FcγRIIIa/CD16a), one inhibitory receptor (FcγRIIb/CD32b), and one glycosylphosphatidylinositol (GPI)-linked receptor, lacking a cytoplasmic tail (FcγRIIIb/CD16b) have been identified (Figure 2) (14, 24–26). These are also described as classical IgG receptors. In addition, non-classical receptors for IgG include two FcR-like receptors, FcRL4/CD307d and FcRL5/CD307e that are homologous to FcγRI, and the receptors hFcRn and hTRIM21. All these receptors, with the exception of FcRL4 (that binds both IgA and IgG) are truly IgG receptors since they do not bind any other class of immunoglobulin (27) (Figure 2).

FcRL4 and FcRL5 are inhibitory receptors that are expressed exclusively on B cells and downregulate B-cell receptor responses (28, 29). FcRL4 is restricted to a subset of memory B cells (30). The hFcRn is a transport receptor that allows IgG recycling. Expression of hFcRn on vascular endothelial cells and on intestinal epithelial cells permits bidirectional IgG transport, from the circulation into tissues and *vice versa*. Also, on placental syncytiotrophoblasts, this receptor allows the transport of maternal IgG into the fetus (20). The hFcRn seems also capable of transporting IgG-bound antigens in dendritic cells (31), macrophages (32), and neutrophils (33), thus promoting antigen presentation and modulating immune responses (21). Similarly to the mouse, the cytosolic receptor hTRIM21 is also ubiquitously expressed (23).

FcγRI is a high-affinity receptor, having three Ig-like extracellular domains. It binds mainly monomeric IgG (34). By contrast, FcγRII and FcγRIII are low-affinity receptors, having two Ig-like extracellular domains. They bind only multimeric immune complexes (34, 35).

Activating receptors are associated with a dimer of the common FcRγ chain, which contains an immunoreceptor tyrosine-based activation motif (ITAM) sequence (Figure 2). An ITAM is a conserved signaling motif with the consensus sequence YxxI/Lx_(6–12)_YxxI/L, where x represents any amino acid (36). Exceptions to this rule are the human FcγRIIa and FcγRIIc, which contain their own ITAM within their cytoplasmic tail. By contrast, the inhibitory receptor FcγRIIb contains an immunoreceptor tyrosine-based inhibition motif (ITIM) within its cytoplasmic tail (Figure 2). An ITIM has the consensus sequence I/V/L/SxYxxL/V (37). FcγRIIb negatively regulates various cell functions including antibody production by the B cell (38), proliferation, degranulation, and phagocytosis in other leukocytes when it is crosslinked with activating FcγRs (37, 39). Most immune cells express both activating and inhibitory FcγRs, hence simultaneous crosslinking establishes a threshold for cell activation (40, 41) that maintains a balanced immune response (42, 43). The GPI-linked receptor FcγRIIIb is expressed mainly on neutrophils and on a subset of basophils (44). It is classified as an activating receptor, although it is not associated with the common FcRγ chain (34). In fact, no other subunits are known to associate with it, and its signaling mechanism remains unknown (4, 14). The human FcγRIIa and FcγRIIIb are exclusive receptors that are not found in other species (24, 45).

### Polymorphisms and Links to Disease Susceptibility

In addition, there are several polymorphisms in the human FcγRII and FcγRIII (46). Two alleles of the gene coding for FcγRIIa generate two isoforms with different aminoacids at position 131. These are known as low-responder (H_131_) and high-responder (R_131_) (47). The H_131_ and R_131_ isoforms are expressed differentially in Caucasian and Asian people (48). For FcγRIIIa also allelic variants exist expressing either valine or phenylalanine at position 158 (49, 50). Similarly, for FcγRIIIb on neutrophils, two isoforms exist differing at four positions, NA1 (R36 N65 D82 V106) and NA2 (S36 S65 N82 I106) (51), and with different glycosylation patterns (52). These differences affect the capacity of FcγRIIIb to interact with human IgG. Therefore, neutrophils from individuals who are homozygous for the NA1 allele have better phagocytosis of IgG-opsonized targets than do neutrophils from NA2-homozygous individuals (53, 54). Also, a point mutation (A78D) in the NA2 allele generates another FcγRIIIb isoform named SH (55). In addition, the gene for FcγRIIIb may be present in a variable number of gene copies in different individuals. Thus, a single person may express all three FcγRIIIb isoforms (56). Several of these polymorphisms have been associated to autoimmune and infectious diseases. FcγRIIa R_131_ has been associated to nephropathy (57), bacterial infections (58), and systemic lupus erythematosus (SLE) (57, 59). FcγRIIIa F_158_ has been associated to SLE (49) and to rheumatoid arthritis (60). FcγRIIIb NA1 has been associated to Wegener granulomatosis (61) and systemic vasculitis (62), while FcγRIIIb NA2 has been associated to SLE in Japanese people (54). These multiple FcγR and their allelic variants vary greatly in their affinity for different IgG classes (35).

### Cell Expression of FcγRs

Fcγ receptors are found on many cells of the immune system (34). The expression pattern of these receptors on the different immune cell types has been recently reexamined with support from new FcγR-specific monoclonal antibodies (Table 1). FcγRI is expressed on monocytes, macrophages, dendritic cells (25), and interferon-γ (IFN-γ)-stimulated neutrophils (63) and mast cells (64). FcγRIIa is expressed on macrophages, neutrophils, mast cells, eosinophils, and platelets. FcγRIIb is expressed on B cells (65), basophils (66), tissue macrophages, dendritic cells (65), and on a small fraction of monocytes and neutrophils (67). FcγRIIIa is expressed mostly on NK cells and weakly on monocytes, macrophages, basophils, and mast cells (26, 34). FcγRIIIb is expressed on neutrophils and by a subset of basophils (44). Interestingly, the expression of some of these classical FcγRs has been found on cells other than hematopoietic cells (68). For example, FcγRI expressed on sensory and motor neurons allows uptake of IgG and release of neurotransmitter (69), while FcγRIIb is expressed on hippocampal neurons (70), and also on liver endothelial sinusoidal cells (71). Thus, FcγR-mediated functions may not always be related to immune cells.

**Table 1:** **Cell expression pattern of Fcγ receptors (FcγRs)**.

	FcγRI	FcγRIIa	FcγRIIb	FcγRIIIa	FcγRIIIb
**Human cell**					
Neutrophil	+^a^	+	+/−^b^	–	++
Monocyte	+	+	+/−^b^	+^c^	–
Macrophage	+	+	+	+^c^	–
B cell	–	–	++	–	–
T cell	–	–	–	–	–
NK cell	–	–	++	++	–
Dendritic cell	+	+	+	–	–
Mast cell	+^a^	+	–	+^c^	–
Basophil	–	+	–	+^c^	+^b^

	**FcγRI**	**FcγRIIb**	**FcγRIII**	**FcγRIV**	

**Mouse cell**					
Neutrophil	–	+	+	+	
Monocyte	+	+	+	+^d^	
Macrophage	+	+	+	+	
B cell	–	+	–	–	
T cell	–	–	–	–	
NK cell	–	–	+	–	
NKT cell	–	–	+	–	
Dendritic cell	+	+	+	–	
Mast cell	–	+	+	–	
Basophil	–	+	+	–	

*^a^Inducible expression*.

*^b^Small subset*.

*^c^Weak expression*.

*^d^LybC low*.

It is worth mentioning that FcγR expression is not fixed and can be altered by other factors. For example, Th1-type cytokines such as IFN-γ and the anaphylatoxin C5a upregulate activating FcγRs expression and downregulate FcγRIIb expression (72, 73), whereas Th2-type cytokines, such as interleukin (IL)-4, IL-10, and transforming growth factor-beta upregulate FcγRIIb expression (41, 74).

### Soluble FcγRs

Another interesting characteristic of FcγRs is that soluble forms exist. They are generated by enzymatic cleavage of membrane-associated receptors or by alternative splicing of the transmembrane region encoding exons. In the first case, these soluble receptors comprise the extracellular part of the receptor, and in the second case they include the extracellular region linked to the intracytoplasmic part of the receptor. Soluble FcγRs are found in serum (75, 76), human saliva (77), and their levels depend on the immune status of the host (78). Recombinant soluble FcγRs bind mouse and human IgG subclasses with a binding profile identical to the corresponding membrane-associated receptors and present immunomodulatory properties (79). Thus, FcγRs present a dual role in immunity. They are signal transduction units for antibodies during activation of leukocytes, and also function as regulatory molecules when produced in solution.

Soluble forms of FcγRs were first described for the mouse FcγRIIb on activated B cells (80), T cells (81), and on fibroblasts expressing a recombinant form of this receptor (80). In murine macrophages (P388D1 cell line), a soluble form of FcγRIIb was detected in tissue culture supernatants. This soluble receptor corresponded to an mRNA derived from the FcγR gene by splicing exons encoding the transmembrane and intracytoplasmic domains (82). Interestingly, B cells, which do not splice the IC1 exon, do not secrete this soluble FcγRIIb isoform (81, 83). The mouse FcγRIII has also been shown to be released in soluble form from activated NK cells (83), macrophage cell lines (82), and Langerhans cells (84).

In human cells, an mRNA splice form of FcγRII without the transmembrane region was detected by PCR in erythroleukemia (K562) and monocytic (U937) cell lines (47, 76). This soluble isoform of FcRII has been found in serum (85), and can also be released from Langerhans cells (86). In addition, a soluble FcγRIIb produced by proteolytic cleavage of the membrane-bound receptor, is released from activated B cells (87, 88). For human FcγRIII, both isoforms, FcγRIIIa and FcγRIIIb are released by proteolytic cleavage upon NK cell (89, 90) and neutrophil activation (91, 92), respectively, by various stimuli. The soluble FcγRIII is found in serum (92), in synovial fluid and saliva (77). No soluble FcγRI isoform has been reported. However, one human FcγRI gene has a stop codon at the 3′ end of the exon coding for the second extracellular domain. Thus, this gene would code for a predictive soluble low-affinity FcγR. Such a secreted receptor has not been identified (79).

The shedding of FcγRIIIa involves mainly matrix metalloproteinases (93), whereas FcγRIIIb is released by the action of both metalloproteinases and serine proteases (94, 95). Metalloproteinase inhibitors mostly blocked phorbol-12-myristate-13-acetate (PMA)-induced, but not cytochalasin B + fMLF-induced shedding of FcγRIIIb. By contrast, serine protease inhibitors mostly blocked cytochalasin B + fMLF-induced, but not PMA-induced shedding of FcγRIIIb (96). Thus, distinct types of proteolytic enzymes seem to be involved in the stimulus-induced shedding of FcγRIIIb from human neutrophils. Because, inhibitors of metalloproteinase members of the A Disintegrin And Metalloproteinase (ADAM) family appeared most efficient in preventing FcγRIIIb shedding (96), more recently it has been shown that ADAM17 is the primary protease mediating FcγRIIIb cleavage (97). ADAM17 is also involved in releasing FcγRIIIa from activated NK cells (97–100). However, in these cells, membrane-type 6 matrix metalloproteinase may also participate in FcγRIIIa shedding (101). FcγRIII presents a short membrane proximal cleavage region where three separate cleavage sites have been identified at positions alanine195/valine196, valine196/serine197, and threonine198/isoleucine199 (102).

Functions for soluble FcγRs are not completely known. However, because their levels in serum depend on the immune status of the host, these soluble receptors have a potent immunomodulatory role (78). In mouse, activation of the immune system by protein antigens such as ovalbumin and parasitic infections increases the levels of soluble FcγRs in serum (103), and in tumor-bearing animals (75). T cell-produced soluble FcγRs inhibited IgM and IgG production (79), and primary and secondary responses were inhibited by recombinant soluble FcγRII both *in vitro* (79) and *in vivo* (81, 83). Also, the intraperitoneally administration of these recombinant soluble receptors inhibited B cell responses induced *via* the B cell receptor, or B cell proliferation induced by mitogens (104). Purified soluble human FcγRIIIb inhibited IgM and IgG production by peripheral blood leukocytes stimulated with pokeweed mitogen (79, 105).

Despite a clear immunomodulatory role for these soluble FcγRs, a potential function for them in immunological disorders has been difficult to demonstrate. However, several examples exist where soluble FcγRs clearly change in pathological conditions. In patients with paroxysmal nocturnal hemoglobinuria, an acquired defect of hematopoietic stem cells in the synthesis or attachment of GPI-anchored proteins, a reduced expression of FcγRIIIb on neutrophils (106), and a reduced level of soluble FcγRIIIb (91, 92) have been reported. The impact for the deficiency of both membrane and soluble FcγRIIIb on the immunological disorders associated with this disease has not been established (79). In patients with multiple myeloma, a reduction of soluble FcγRIIIb correlated with disease severity (79). This reduction was associated with a slight decrease in circulating neutrophils, but not with a significant defect in soluble FcγRIIIb production by neutrophils, as detected *in vitro* (107). Treatment of acute immune thrombocytopenic purpura (ITP) with intravenous immunoglobulin (IVIG) induces partial or complete responses, shown by increases in platelet count. The mechanism for this clinical benefit may be the blockade of FcγRs. Platelets sensitized by IgG could not be cleared by cells of the reticuloendothelial system if their FcγRs were blocked with IVIG (108, 109). In the same way, children with ITP, who were treated with intravenous infusions of Fc fragments of IgG, showed rapid increases in platelet counts together with partial or complete responses (110). In addition, an increase in serum soluble FcγRIII concentration correlated with the rise in platelet count (110). Thus, it seems that FcγR blockade is the main mechanism of action of IVIG in ITP. However, other immunoregulatory mechanisms triggered by the presence of increased soluble FcγRIII could also be involved in the clinical benefit observed during ITP treatment (110). Also, in human immunodeficiency virus (HIV)-infected patients, a reduction of soluble FcγRIII levels in serum was reported. The reduction of soluble receptor correlated with a reduction of CD4^+^ T cells (111). Although, no specific changes in the number of NK cells expressing FcγRIIIa were found in this study, recently it has been proposed that NK cell activation during HIV infection leads to profound decreases in FcγRIIIa expression on NK cells (112). These results suggest that NK cell activation-induced FcγR cleavage may result in the soluble FcγRIII that associates with HIV disease progression, further suggesting a linkage between chronic NK cell activation and HIV disease progression (112).

More research on the role of these soluble FcγRs in various immunological and inflammatory disorders is needed, in order to fully understand their effects on the immune response and to use them in novel therapeutic approaches.

## IgG Binding Affinity for FcγRs

As described above, there is one high-affinity Fcγ receptor, FcγRI (CD64), and two groups of low-affinity FcγRs, FcγRII and FcγRIII. The FcγRII group includes FcγRIIa, FcγRIIc, and FcγRIIb (CD32a, CD32c, and CD32b), while the FcγRIII group includes FcγRIIIa and FcγRIIIb (CD16a and CD16b). This means that a single IgG molecule cannot bind to most FcγRs. On the contrary, antigen-antibody (immune) complexes promote many low-affinity interactions between FcγR and IgG. In consequence, only immune complexes are able to induce the crosslinking of FcγR on the membrane of immune cells leading to the various antibody-mediated cell functions (Tables 2 and 3).

**Table 2:** **Relative affinities of human IgG subclasses for human Fcγ receptors (FcγRs)**.

	FcγRI	FcγRIIa		FcγRIIb	FcγRIIc	FcγRIIIa		FcγRIIIb
		H_131_	R_131_			V_158_	F_158_	
**IgG subclass**								
IgG1	+++	++	++	+	++	++	++	++
IgG2	–	++	+	+	+	++	+	–
IgG3	+++	++	++	+	++	++	++	++
IgG4	+++	++	++	+	++	++	+	++

*+++, high affinity; ++, low affinity; +, very low affinity; –, no binding*.

**Table 3:** **Relative affinities of mouse IgG subclasses for mouse Fcγ receptors (FcγRs)**.

	FcγRI	FcγRIIb	FcγRIII	FcγRIV
**IgG subclass**				
IgG1	–	++	+	–
IgG2a	+++	++	++	+++
IgG2b	++	++	++	+++
IgG3	+	–	–	–

### The Role of Particular IgG Subclass

Because, there are four subclasses of IgG (IgG1, IgG2a, IgG2b, and IgG3 in mice; and IgG1, IgG2, IgG3, and IgG4 in humans) (113), different kinds of immune complexes exist. It has been observed in many *in vivo* studies that the different IgG subclasses indeed can activate different cell responses. For example, in mice, IgG2b was better at eliminating B cells (114) and T cell lymphomas (115) than IgG1. Also, anti-erythrocyte antibodies of IgG2a and IgG2b subclasses were better in mediating phagocytosis of opsonized erythrocytes than antibodies of IgG1 and IgG3 subclasses (116, 117). In addition, IgG2a could induce a more severe glomerular inflammation than IgG2b, and in turn IgG2b could do it better than IgG1 (118).

All these reports confirmed that different IgG subclasses mediate different cellular responses *in vivo* and have suggested that these different cellular activities result from crosslinking different FcγRs. In consequence, a great interest exists for determining which type of IgG binds to which FcγR and what particular receptor is involved in mediating a certain cellular function. In humans, it was shown that most FcγRs bind primarily IgG1 and IgG3 over the other subclasses of IgG (Table 2). Similarly, in mice it was shown that IgG1 binds only to mFcγRIII, while IgG2a binds to all types of activating FcγR. IgG2b binds to mFcγRIII and mFcγRIV. IgG3 does not seem to bind significantly to any of the FcγR (14, 24, 117, 119) (Table 3).

In agreement with these data, IgG1 activity was lost in mice deficient in mFcγRIII (117, 120). For IgG2a and IgG2b, however, the correlation with particular FcγRs is not as simple. In some model systems, the activity of these IgG classes was lost in mFcγRIII-deficient mice, while it was not in others (13). Therefore, it seems clearly established that different IgG subclasses mediate different cellular responses by crosslinking different FcγRs. However, the mechanism used to generate this IgG–FcγR selectivity is not completely understood.

Obviously, this selectivity depends mainly on the affinities of different IgG subclasses to particular FcγRs. For this reason, detailed studies to measure the affinities of IgG classes to the various FcγRs have been conducted both for mice FcγRs (117) and more recently for all human FcγRs (35). Through these studies, it was found that murine IgG1 has higher affinity for the inhibitory FcγRIIb than for the activating mFcγRIII. By contrast, murine IgG2a and IgG2b have higher affinity for the activating mFcγRIV than for the inhibitory mFcγRIIb. These results suggest that for IgG1 a high threshold for activation exists, while for IgG2a a lower threshold for activation is present, and also help explain why in most *in vivo* responses IgG2a antibodies seem to be much more potent and effective (121, 122). In the case of humans, it was found that IgG1 and IgG3 bind to all FcγRs. IgG2 binds mainly to FcγRIIa (H_131_ isoform) and FcγRIIIa (V_158_ isoform), but not to FcγRIIIb (35). IgG4 binds to many FcγRs (35). Thus, it is clear that different IgG subclasses engage different FcγRs depending on the relative affinity of these receptors for a particular IgG class (24).

### The Role of Antibody Glycosylation Pattern

All IgG antibodies have one carbohydrate (sugar) side chain added to asparagine 297 (Asp^297^) in their Fc portion. This *N*-glycosylated carbohydrate side chain is important for IgG function (123) and its deletion leads to poor binding to FcγRs (124). The N-glycans attached to the Fc portion of the IgG molecule are heterogeneous in their sugar composition (15). The heterogeneous pattern of glycosylation may contain sugar residues such as galactose, fucose, and sialic acid in straight or branching patterns (16). This heterogeneous pattern may also change with age and disease (125). For example, terminal galactose and sialic acid residues were reduced in active autoimmune disease (126, 127), while they were increased during pregnancy (128, 129). These changes in the glycosylation pattern seem to regulate IgG activity (130).

Many IgG antibodies present a fucose residue linked to an *N*-acetylglucosamine residue (131). The absence of this fucose residue increased the binding affinity of antibodies to human FcγRIIIa and its mouse ortholog mFcγRIV (132). Together with the increased receptor binding, these IgG antibodies also augmented ADCC activity against various tumor cells (119, 132, 133). These observations have lead to producing recombinant IgG antibodies with low fucose levels in order to increase their ADCC activity. Several of these antibodies are now in clinical trials to test their therapeutic potential (134).

IgG antibodies also have sugar side chain often terminating with sialic acid residues (135). High levels of terminal sialic acid correlate with very low affinity for FcγRs and also with reduced ADCC activity (127, 136). These sialic acid-rich antibodies were also found to preferentially bind other cellular receptors different from FcγRs. Specific ICAM-3 grabbing non-integrin-related 1 and its human ortholog dendritic cell specific ICAM-3 grabbing non-integrin were identified as receptors for sialic acid-rich IgG (137). Hence, terminal sialic acid can modify IgG activity by promoting less binding to FcγRs and more binding to other novel (type II) antibody receptors (17, 138).

## Fc Receptor Signaling

**Figure 3:**
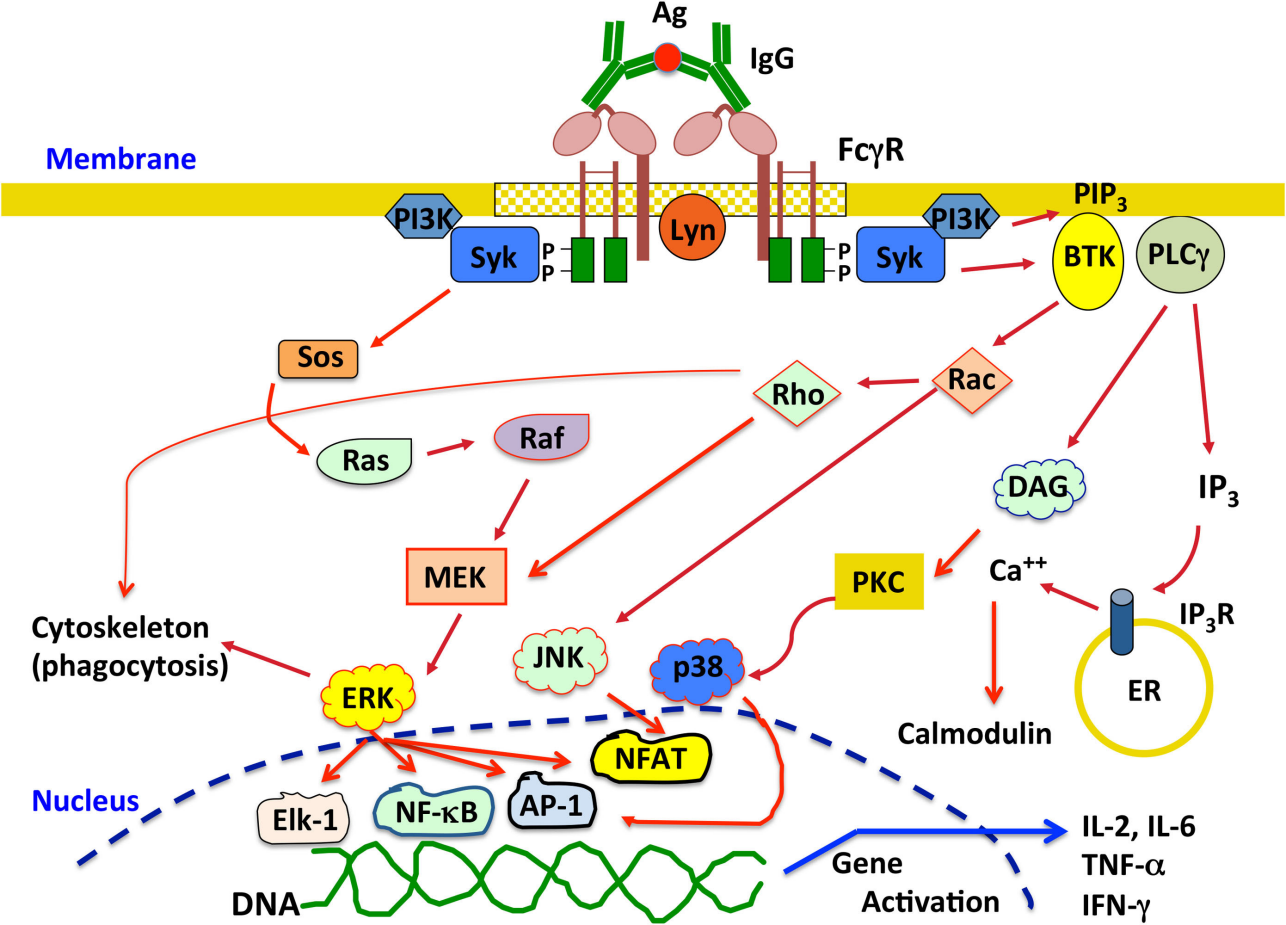
**Overview of the signaling pathways activated upon crosslinking activating Fcγ receptors (FcγRs)**. Engagement of activating FcγRs by IgG immune complexes induces receptor crosslinking and phosphorylation of tyrosine residues in the immunoreceptor tyrosine-based activation motif domains by Src family kinases, for example, Lyn. Phosphorylated tyrosines then become docking sites for Syk, which in turn phosphorylates multiple substrates leading to different signaling pathways that ultimately activate various cell responses. See text for details. P represents a phosphate group; Ag, antigen; Syk, spleen tyrosine kinase; MEK, MAPK/ERK kinase; ERK, extracellular signal-regulated kinase; PI3K, phosphatidylinositol-3 kinase; PIP3, phosphatidylinositol 3,4,5-trisphosphate; BTK, Bruton’s tyrosine kinase; JNK, c-Jun N-terminal kinase; PLCγ, phospholipase Cγ; DAG, diacylglycerol; IP_3_, inositol triphosphate; PKC, protein kinase C; ER, endoplasmic reticulum; NFAT, nuclear factor of activated T cells; IL-2, interleukin-2; IL-6, interleukin-6; TNF-α, tumor necrosis factor α; IFN-γ, interferon-γ.

All activating FcγR containing ITAM sequences seem to signal in a similar way at least at the first signaling step. After crosslinking of activating FcγRs, Src family kinases, such as Fyn, Lck, or Lyn, get activated followed by activation of Syk (spleen tyrosine kinase) family kinases. These kinases phosphorylate tyrosines within the ITAM. Phosphorylated tyrosines then become docking sites for Syk, which in turn phosphorylates multiple substrates leading to different cell responses (4, 14, 139). The Ras pathway can be activated through phosphorylation of Sos. This leads to activation of Ras, which in turn phosphorylates Raf, leading to activation of MAPK/ERK kinase (MEK) and extracellular signal-regulated kinase (ERK). This pathway is associated with activation of transcription factors such as AP-1, nuclear factor of activated T cells (NFAT), and NF-κB that control cytokine production and expression of cell survival proteins (Figure 3). Syk can also induce activation of phosphatidylinositol-3 kinase, which produces phosphatidylinositol 3,4,5-trisphosphate (PIP3). This phospholipid recruits pleckstrin homology domain-expressing proteins such as Bruton’s tyrosine kinase and other Tec family kinases involved in activation of small GTPases, such as Rho and Rac that are required for cytoskeleton remodeling. These small GTPases also impinge in activation of MEK and c-Jun N-terminal kinases, leading to nuclear factor activation (Figure 3). PIP3 also recruits phospholipase Cγ, which in turn generates diacylglycerol (DAG) and inositol triphosphate (IP_3_). DAG activates PKC (protein kinase C), an important serine/threonine kinase that can lead to activation of the MAP kinases ERK and p38 (Figure 3). IP_3_ induces release of intracellular calcium from the endoplasmic reticulum. Calcium regulates several proteins such as calmodulin and calcineurin, which are important for activation of some nuclear factors like NFAT (Figure 3). Activation of different nuclear factors induces expression of cytokines important for inflammation and immune regulation, such as IL-2, IL-6, IL-8, IL-10, tumor necrosis factor α (TNF-α), and IFN-γ (140–142) (Figure 3).

The signal transduction pathways activated by FcγRs binding to high avidity immune complexes, induce multiple cell responses including phagocytosis, respiratory burst, cytokine and chemokine production, and antibody-dependent cell-mediated cytotoxicity (ADCC) (14, 18, 41). The particular signaling molecules activated to initiate each cell response are not clearly defined in part because every cell has more than one type of FcγR and all receptors can bind more than one type of IgG. Thus, it is not clear whether each receptor leads to a particular response or the average signaling from various receptors activates a predetermined cell response. As discussed later in more detail, recent research is beginning to shade light into this issue.

## Each FcγR Leads to Unique Cellular Responses

As discussed above, it is now clear that different IgG subclasses engage different FcγRs to induce particular cellular responses *in vivo*. However, the data published so far does not explain what cell function is activated in response to a particular type of Fcγ receptor. We can think of at least two mechanisms to generate this IgG–FcγR response selectivity: in one, each immune cell is already programmed to perform a particular cell function after FcγR crosslinking, independently of the receptor used. This does not seem likely because as mentioned before, each type of immune cell can give different responses depending on the class of IgG and also on the conditions the cell encounters (such as inflammation, etc.) (42, 72). In the second mechanism, each FcγR activates a particular signaling pathway leading to a unique cell response. This mechanism is supported by recent reports where individual FcγR were crosslinked on human neutrophils (143–147).

**Figure 4:**
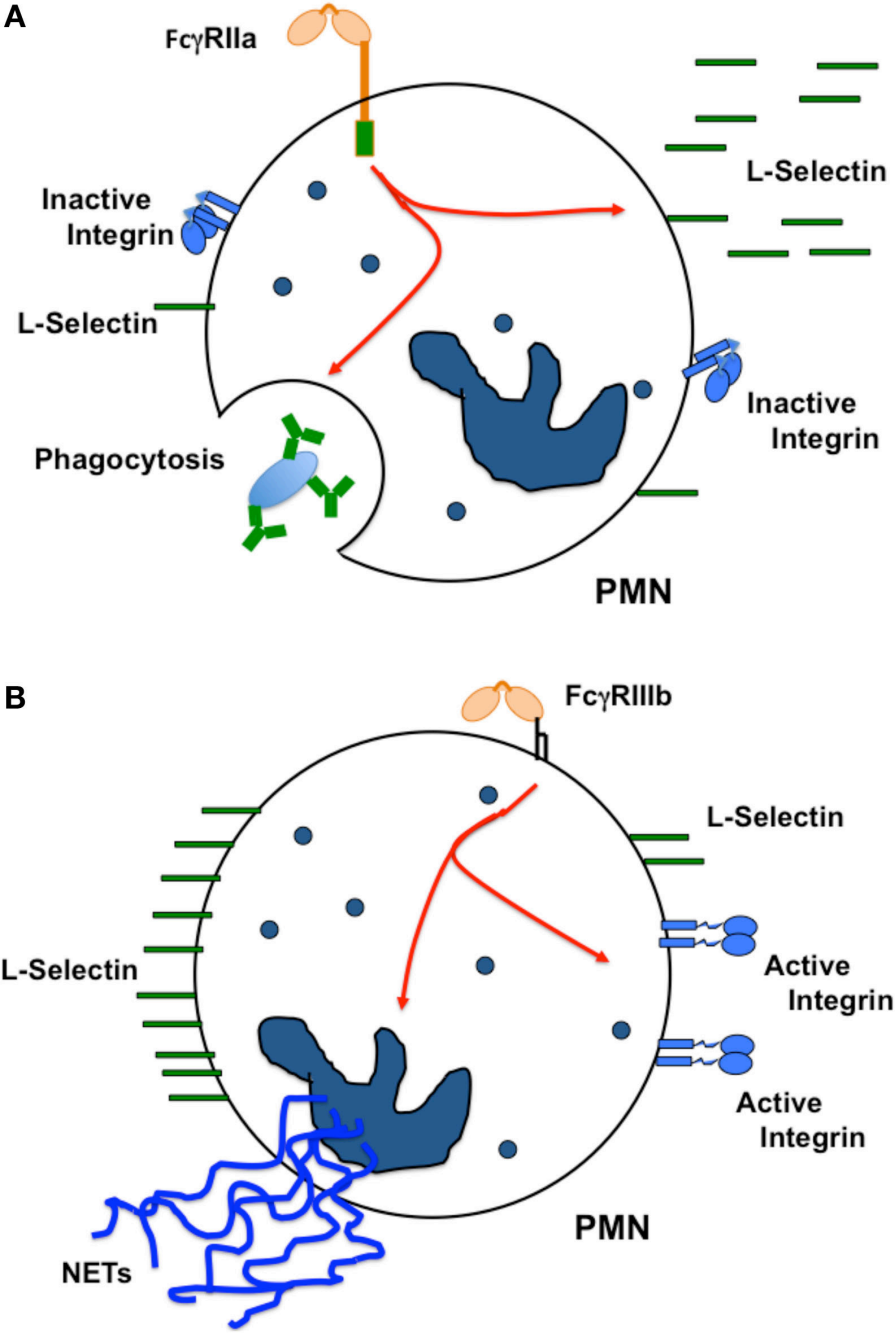
**Each FcγR induces particular cellular responses**. In human neutrophils (PMN), **(A)** FcγRIIa causes L-selectin shedding from the cell membrane, and also activates efficient phagocytosis. By contrast, **(B)** FcγRIIIb does not cause L-selectin shedding, but stimulates activation of β1 integrins promoting in this way a proadhesive phenotype. FcγRIIIb also induces formation of neutrophil extracellular traps (NETs). The oval represents an IgG-opsonized particle.

Human neutrophils express only two FcγRs, FcγRIIa and FcγRIIIb (45). These receptors are different in the way they are anchored to the cell membrane. FcγRIIa has a typical transmembrane and cytoplasmic tail containing an ITAM for signaling. By contrast, FcγRIIIb is a GPI-linked receptor, lacking a cytoplasmic tail, and its signaling mechanism remains unknown. The first report suggesting that these receptors could initiate distinct cellular responses came out over 20 years ago. It was reported that both FcγRs were capable of signaling, but while FcγRIIIb induced actin polymerization in a Ca^2+^-dependent manner, FcγRIIa did not (148). This pioneer work did not manage to maintain the idea of one receptor one response. However with time other reports have provided new evidence that supports this idea. For example, it was later reported that FcγRIIa, but not FcγRIIIb could induce an increase in L-selectin expression (149). Based on this, it was suggested that FcγRIIIb-mediated activation of circulating neutrophils could lead to a proadhesive phenotype (149). Supporting this view, it was also found that after selective engagement of each receptor with specific monoclonal antibodies, FcγRIIIb, but not FcγRIIa, was able to activate β1 integrins (143). This activation was not due to an increase in integrin expression but rather to an increase in binding affinity for integrin ligands such as fibronectin (143). By contrast, when the major cell response of neutrophils, arguably phagocytosis (150, 151), was examined with receptor specific opsonized beads, FcγRIIa was the predominant FcγR mediating this response. FcγRIIIb contribution to phagocytosis was minimal (145). Thus, at least in human neutrophils each Fcγ receptor is used to activate unique cell responses. FcγRIIa induces mainly phagocytosis, while FcγRIIIb promotes an adhesive phenotype *via* activation of β1 integrins (Figure 4).

**Figure 5:**
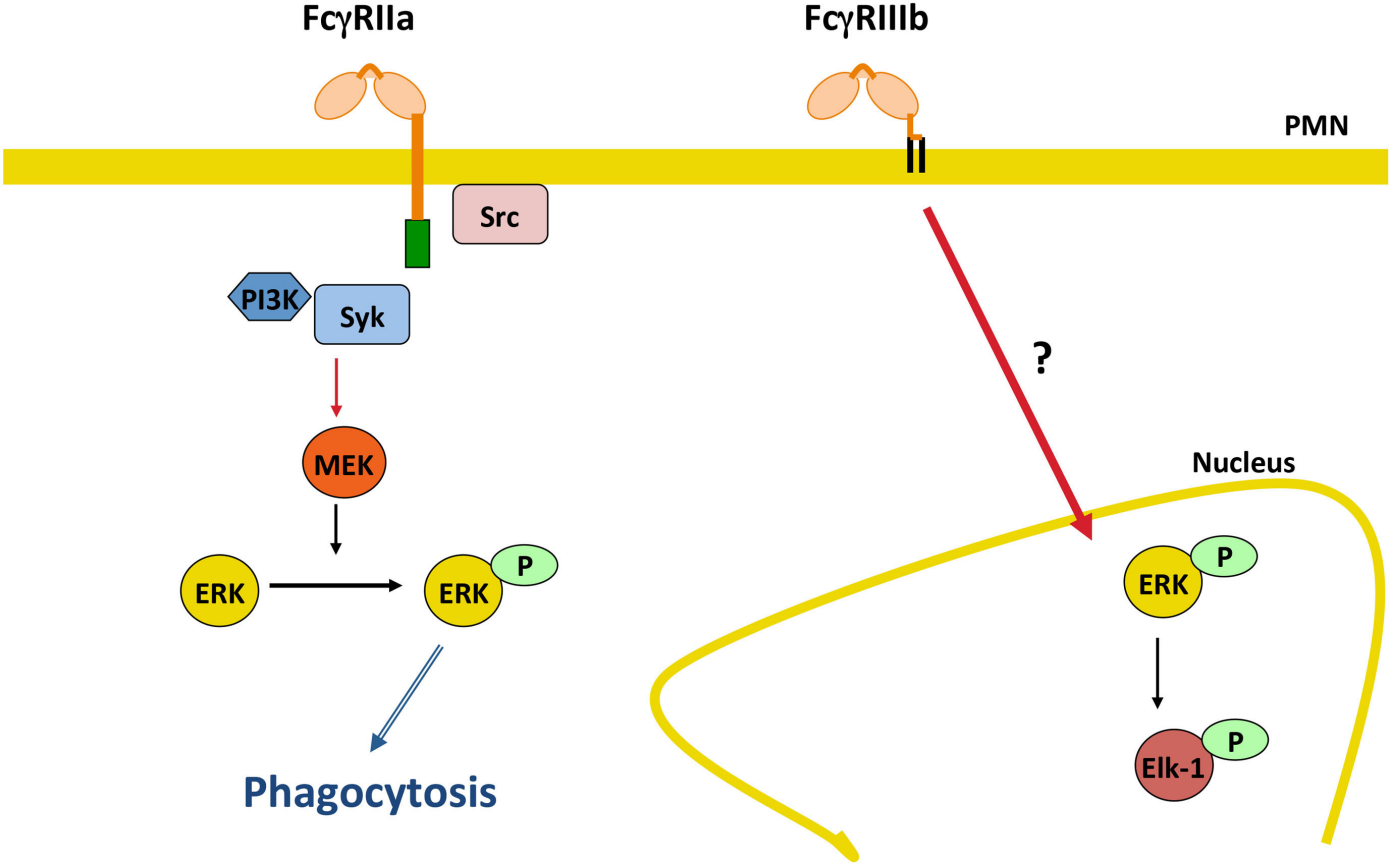
**FcγRIIIb initiates signaling pathways to the nucleus**. In human neutrophils (PMN), FcγRIIa activates the classical immunoreceptor tyrosine-based activation motif-mediated signaling pathway leading to efficient phagocytosis. By contrast, FcγRIIIb induces a robust increase in phosphorylated ERK in the nucleus, and also efficient phosphorylation of the nuclear factor Elk-1. The FcγRIIIb signaling pathway remains a mystery and further future research is needed in this area. P represents a phosphate group; Syk, spleen tyrosine kinase; PI3K, phosphatidylinositol-3 kinase; MEK, ERK kinase; ERK, extracellular signal-regulated kinase.

In addition, it was recently found that FcγRIIIb signaling to the neutrophil nucleus was much more efficient than FcγRIIa signaling. FcγRIIIb, but not FcγRIIa, promoted a robust increase in phosphorylated ERK in the nucleus, and also efficient phosphorylation of the nuclear factor Elk-1 (144) (Figure 5). Interestingly, FcγRIIa also induced phosphorylation of ERK in the cytosol (144, 152), but this active ERK seems to function mainly in enhancing phagocytosis and not in nuclear signaling (Figure 5). An important point still unresolved is the actual FcγRIIIb signaling pathway. For FcγRIIa, the FcγR signaling pathway resembles the classical ITAM-mediated pathway (4, 14, 139, 153) (Figure 3), while for FcγRIIIb, the signaling pathway remains a mystery and further research is needed in this area (Figure 5).

**Figure 6:**
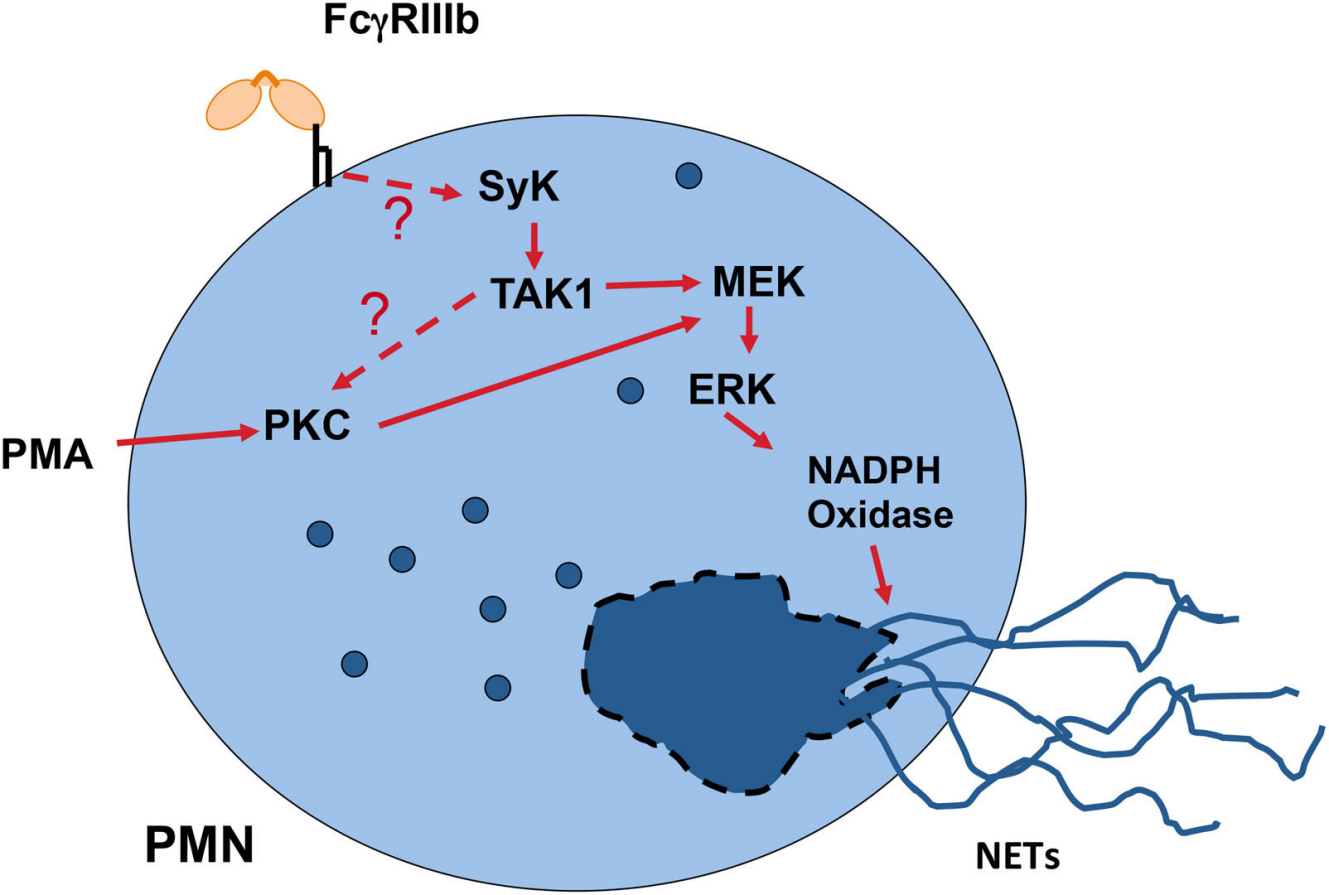
**Model for FcγRIIIb signaling to induce neutrophil extracellular trap (NET) formation**. In human neutrophils (PMN), crosslinking FcγRIIIb results in activation of Syk (spleen tyrosine kinase) and TAK1 (transforming growth factor-β-activated kinase 1). These kinases lead to activation of PKC (protein kinase C) and the MEK/ERK signaling pathway to generate the production of reactive oxygen species *via* NADPH oxidase and to induce NETs formation. The question marks indicate that the mechanism for FcγRIIIb-induced Syk activation is not known, and that it is not clear whether TAK1 functions upstream of PKC. Model based on Alemán et al. (161).

Another important cellular function of neutrophils to kill microbes is the formation of neutrophil extracellular traps (NETs) (154, 155). These structures are induced by several pathogens, including virus, bacteria, fungi, and parasites (156). Also, pro-inflammatory stimuli such as lipopolysaccharide, TNF-α, and PMA are efficient inducers of NETs (157). Because, antigen-antibody complexes are also capable of inducing NET formation (158, 159), it was clear that FcγRs were involved in NET formation. Recently, it was reported that FcγRIIIb is the receptor responsible for NET formation in response to immobilized immune complexes (160). In this study, NET formation induced by immobilized immune complexes was blocked by antibodies against FcγRIIIb, but not by antibodies against FcγRIIa (160), indicating that solely FcγRIIIb mediates NET release. Moreover, by direct crosslinking of each type of FcγR with specific monoclonal antibodies it was also confirmed that only FcγRIIIb is capable of inducing NET formation (147, 161). Although, the initial signaling mechanism for FcγRIIIb remains unknown, the signaling pathway for this cell response has been shown to involve the Syk and TAK1 kinases, as well as the MEK/ERK cascade (Figure 6) (161). Because FcγRIIIb is a GPI-linked receptor it is not clear how it can connect to the ERK pathway. However, it is known that GPI-linked proteins concentrate in lipid rafts on the cell membrane. In these rafts many signaling molecules such as Src family tyrosine kinases concentrate, and it is possible that FcγRIIIb upon ligand binding can connect somehow with these kinases and activate Syk. A possible mechanism is the binding of the receptor, within the lipid rafts, to a putative ITAM-containing molecule (151). After Syk activation, a signaling molecular complex can be organized leading to activation of other kinases such as TAK1 (Figure 6). Many steps are still unknown and future research will help in elucidate this signaling pathway.

Taken together, these reports strongly support the hypothesis that each FcγR is capable of initiating particular signaling pathways that lead to unique cell responses. This information would certainly be very helpful in the future for controlling some of the cellular responses in clinical settings. For example, during a strong infection efficient phagocytosis may be desirable. Considering that IgG2 displays a stronger binding to FcγRIIa than to FcγRIIIb (35) (Table 2), one could predict that antibodies of the IgG2 subclass would be much better at inducing phagocytosis by neutrophils. Thus, inducing the production of IgG2 antibodies against certain pathogens, would very likely improve the phagocytosis response against them. Following the same idea, new monoclonal antibodies against tumors have been developed for recognition of malignant cells. Because on NK cells the only activating Fcγ receptor is FcγRIIIa, finding antibodies with better binding (higher affinity) to FcγRIIIa should improve the activation of ADCC. Indeed, this has been shown to be the case for several anti-tumor antibodies (12, 162, 163). This means that, when we know what is the particular cellular response initiated by each FcγR on an immune cell, we could find ways to improve the IgG binding interaction and enhance the response, or *vice versa* to block the IgG binding interaction and in consequence inhibit the response.

## Conclusion

Fcγ receptors expressed in many immune cells are capable of activating different cellular responses important not only for controlling microbial infections but also for regulating immunity. Different subclasses of IgG antibodies bind the various FcγRs with different affinities. These FcγRs are expressed on a wide variety of leukocytes and are capable of activating when crosslinked with immune complexes, different cellular responses of great importance for host defense and for immune regulation. Recent evidence suggests that a specific Fcγ receptor activates particular cell responses. At least for the human neutrophil it is clear that FcγRIIa activates efficient phagocytosis, while FcγRIIIb signals to the nucleus for nuclear factor activation and NETs formation. Therefore, in principle, a particular cell response could be induced or inhibited by engaging or blocking the corresponding FcγR. For example, using IgG2 antibodies a better phagocytosis response should be generated in neutrophils. Because, FcγRs are responsible not only of initiating anti-microbial responses, but also of controlling the intensity of the immune response, there is growing interest in revealing what specific Fcγ receptor activates a particular cell response. Information similar to the one described for neutrophil FcγRs on other immune cells, such as monocytes or dendritic cells, is not available. We will certainly see in the near future much more research in this area.

## Author Contributions

CR conceived the issues which formed the content of the manuscript and wrote the manuscript.

## Conflict of Interest Statement

The author declares that the research was conducted in the absence of any commercial or financial relationships that could be construed as a potential conflict of interest.
